# Position tracking control of electro-hydrostatic actuators via adaptive finite-time backstepping and state observation

**DOI:** 10.1038/s41598-026-44019-0

**Published:** 2026-03-21

**Authors:** Van Du Phan, Hung Le Nguyen, Van Chuong Le, Kyoung Kwan Ahn

**Affiliations:** 1 School of Engineering and Technology, Vinh University, Nghe An, 43100 Vietnam; 2 School of Mechanical Engineering, University of Ulsan, Ulsan, 44610 South Korea

**Keywords:** State observer, Finite-time convergence, Backstepping control, Neural network, Engineering, Mathematics and computing, Physics

## Abstract

In this paper, the adaptive finite-time backstepping control approach is proposed for electro-hydrostatic actuator with symmetrical cylinder (EHSC) under the unmeasured states and disturbances. The state observer (SOB) is constructed to estimate the unmeasured states. To address the unknown dynamic system during design control procedure, the adaptive law based on the maximum norm of the radial basis function neural network (NN) is introduced for EHSC. In addition, the adaptive finite-time backstepping control scheme using SOB is synthesized to enrich the tracking performance of the EHSC. It is exhibited by theoretical Lyapunov analysis and backstepping approach that the finite-time stability is guaranteed under unmeasured states and disturbances. Ultimately, both simulation and experiment results are conducted to further confirm the workability of the suggested control algorithm.

## Introduction

For the past few decades, the electro-hydrostatic actuator with a symmetrical cylinder (EHSC) has been widely adopted and is considered a promising solution in various fields, including the automotive industry, construction machinery, marine engineering, and aerospace. These applications demand fast response, high reliability, and precise positioning under external loads^1,2^. However, several adverse factors—such as nonlinearities, external disturbances, and unmeasured states can negatively impact system stability and degrade control performance^3^. It is essential that these challenges be addressed during the control design process. Control strategies such as adaptive control^4^, intelligent control^5^, and robust control^6^ are introduced regarding the above-mentioned problem. To this end, an advanced robust control strategy is proposed to enhance the performance of EHSC systems.

In order to deal with the unknown nonlinear function, neural network technology is a promising tool. Leveraging its learning capabilities, an NN can approximate arbitrary continuous functions^7,8^. In^9^, an NN estimator was integrated into a robust controller to mitigate the effects of disturbances in hydraulic systems. However, the NN in that approach requires full state information, necessitating the use of multiple sensors. In^10^, a NN-based adaptive control was reported to deal with the unknown actuator dynamics of the hydraulic manipulator. The comparative simulations were performed to demonstrate the validity of presented strategy. In^11^, NN with a norm of weighting estimator-based sliding mode control was presented for electro-hydraulic servo-valve-controlled actuators in the presence of uncertainties. In^12^, reinforcement learning-based multilayer neuroadaptive algorithm was developed to address tracking control problems of the uncertain nonlinear systems. However, these prior works generally rely on conventional multilayer NN structures and complex learning algorithms, resulting in high computational burdens. Moreover, the closed-loop systems they produced only guarantee bounded tracking performance in the presence of disturbances, without ensuring finite-time convergence. Therefore, the development of a novel adaptation scheme for NN-based control is urgently needed to enhance performance while reducing the computational complexity of the training process.

To achieve satisfactory performance in EHSC systems, most existing methods rely on data from up to three sensors, rather than using only a position sensor^13,14^. This reliance on additional equipment increases costs under normal operating conditions. In^15^, an output-feedback sliding mode approach was developed for a hydraulic cylinder. Subsequently, a finite-time observer-based robust control scheme was proposed that required only position measurements. However, finite-time convergence was achieved only for the observer and not for the main controller. In^16^, a variable-bandwidth extended state observer-based adaptive controller was proposed for an electro-hydraulic servo systems subject to disturbance and noise. In^17^, a nonlinear unknown input observer was applied to estimate both position and fault signals, despite the presence of disturbances and uncertainties. However, finite-time convergence was not achieved, and experimental validation was not provided. In^18^, an improved disturbance observer was proposed to estimate system states and disturbances in an electro-hydraulic system, aiming to enhance estimation performance. In^19^, The control architecture, integrating state-filtered disturbance rejection, was introduced to address both smooth and non-smooth disturbances. However, the aforementioned methods require detailed hydraulic cylinder models and do not guarantee finite-time convergence.

The backstepping is powerful, and easy for design controller through the Lyapunov function candidate^20–23^. In^24^, the backstepping framework was concerned to construct a controller for EHSC. And performance control obtained as time reaches to infinity. In^25^, the development of a controller using backstepping technique for an electro-hydraulic actuator was reported. The simulation results showed that factors resulting in disturbance and nonlinearities reduced the capacity of the presented controller. In^26^, a backstepping with first-order filter scheme for position tracking of an EHSC was investigated. An adaptation technology was implemented to handle the nonlinear matched disturbance. The comparative experiment indicated that the hybrid backstepping and adaptation technique brings satisfactorily control performance. In^27^, adaptive backstepping with prescribed performance was developed for hydraulic system. All system states were bounded in a preset region. The tracking performance was verified by experiment results. It is noteworthy that the existing work concentrates on the tracking problem with infinite-time stability. In addition, the finite-time control provide a faster convergence time compared to other asymptotic tracking control^28^. Nevertheless, finite-time backstepping control and its effectiveness has remained an open issue for EHSC.

Motivated by the aforementioned conservativeness, this paper proposes a state observer (SOB)-based finite-time backstepping control strategy with an enhanced adaptive law to address the trajectory tracking problem of an EHSC system. A neural network (NN)-based adaptation scheme is employed to approximate the unknown system dynamics of the EHSC. To manage unmeasured states and improve overall system performance, the SOB-based finite-time backstepping controller is designed. In comparison to previous studies, the main contributions of this work can be summarized as follows:

(1) Distinctly different from previous works^9,10^, this study first employs the maximum norm of the neural network (NN) in designing both the SOB and finite-time controller of the EHSC system, effectively addressing the unknown system dynamics while reducing the computational burden during NN training.

(2) An adaptive finite-time backstepping controller is proposed to enhance tracking performance. Additionally, a state observer (SOB) is developed to estimate unmeasured states, making the proposed control approach more practical by requiring only a position sensor, without the need for additional sensors.

(3) The stability of the SOB and the closed-loop control system is verified using the backstepping control approach and Lyapunov principle. Furthermore, simulation and experimental results are presented to validate the effectiveness of the proposed control algorithm as compared with other methods.

This article is structured as follows: In Sect.  2, the EHSC model and some problem statements are presented. The proposed control algorithm and system stability are performed in Sect.  3. The simulation and comparative experimental results are conducted in Sects.  4 and 5, respectively. Ultimately, Sect.  6 highlights the conclusions and future work.

**Fig. 1:**
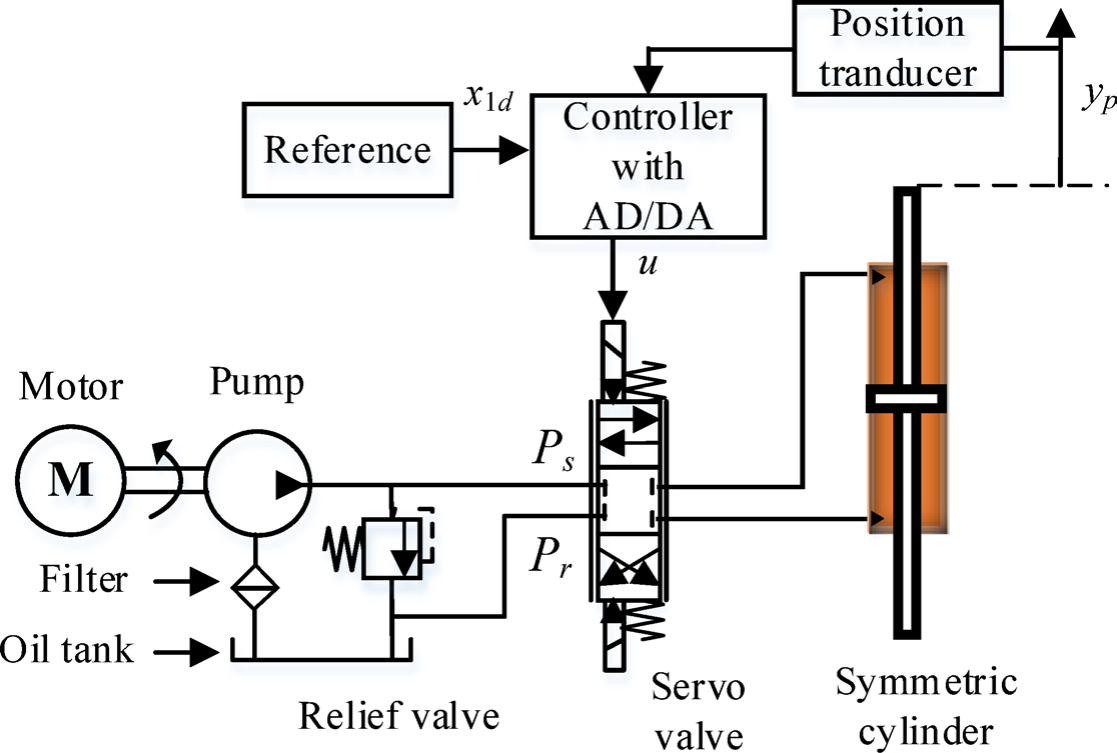
The EHSC structure.

## Problem description

Figure 1 illustrates the comprehensive EHSC, which includes a hydraulic system, a measurement module, and a controller.

According to Newton’s second law, the EHSC’s dynamics are described as follows:

1 $$M{\ddot {y}_p}={p_L}{A_s} - b{\dot {y}_p}+\varphi \left( {t,{y_p},{{\dot {y}}_p}} \right)$$

where *y*_*p*_, *M*, and *b* denote the cylinder’s position, load mass, and viscous damping coefficient; *A*_*s*_ represents effective area of cylinder; $\varphi ( \bullet )$is the disturbance factors; *p*_*L*_ denotes the load pressure.

In the EHSC, a servo valve (SV) is utilized to adjust the direction and flow of oil. It is noteworthy that the spool displacement *z*_*v*_ of SV is linear change with the control action *u* (z_v_ = *k*_*SV*_*u*). Therefore, the characteristic equation expressing the relationship between load flow *Q*_*L*_ and control action *u* is formulated by^18^

2 $${Q_L}={k_t}u\sqrt {{P_s} - sign\left( u \right){p_L}}$$

where *P*_*s*_ denotes the pressure’s source, *k*_*SV*_ is a positive constant.

The load flow *Q*_*L*_ is also provided by^9^:

3 $${Q_L}={A_s}{\dot {y}_p}+\frac{{{V_a}}}{{4{\beta _e}}}{\dot {p}_L}+{C_s}{p_L} - {Q_\Delta }$$

where *C*_*s*_ and *β*_*e*_ are the total leakage term and the Bulk modulus of oil. *V*_*a*_ and *Q*_*Δ*_ define a total volume of the cylinder and uncertainty model.

Defining$\xi ={[{\xi _1},{\xi _2},{\xi _3}]^T} \triangleq {[{y_p},{\dot {y}_p},\frac{{{A_s}{p_L}}}{M}]^T}$ as the EHSC’s state variables. Based on (1) − (3), the total of EHSC dynamic can be represented as follows:

4 $$\left\{ {\begin{array}{*{20}{l}} {{{\dot {\xi }}_1}={\xi _2}} \\ {{{\dot {\xi }}_2}={\xi _3}+{q_1}{\xi _2}+{d_1}} \\ {{{\dot {\xi }}_3}=fu+{q_2}{\xi _2}+{q_3}{\xi _3}+{d_2}} \end{array}} \right.$$

where.

$${q_1}= - \frac{b}{M},{q_2}= - \frac{{4{\beta _e}{A^2}}}{{m{V_c}}},{q_3}= - \frac{{4{\beta _e}{C_0}}}{{{V_c}}},{d_1}=\frac{\varphi }{M},$$

$$f=\frac{{4{A_s}{\beta _e}{k_{SV}}}}{{M{V_a}}}\sqrt {{P_s} - \frac{M}{{{A_s}}}{\xi _3}sign\left( u \right)} ,{d_2}=\frac{{4A{\beta _e}}}{{m{V_a}}}{Q_\Delta },$$

### Assumption 1

The mismatched and matched disturbances *d*_1_ and *d*_2_ are bounded and they satisfy $\left| {{d_i}} \right| \leqslant {D_i};i=1,2$, in which *D*_*i*_, are positive constants.

### Remark 1

The objective of control methodology is to propose an adaptive neural network controller that the EHSC’s position follows the desired position signal as closely as possible.

### Lemma 1

^29^: Considering *k*_0_ > 0, Young’s inequality is given as follows:

5 $${c_1}{c_2} \leqslant \frac{{k_{0}^{m}}}{p}{\left| {{c_1}} \right|^m}+\frac{1}{{qk_{0}^{q}}}{\left| {{c_2}} \right|^q}$$

where $${c_1},{c_2} \in R,m,q>1{\text{ and }}\left( {q - 1} \right)\left( {m - 1} \right)=1$$.

### Lemma 2

^30^: For real-valued function *s*(*x*_1_, *x*_2_) > 0, any positive real numbers *p*, *q*, the following inequalities hold:

6 $${\left| {{x_1}} \right|^p}{\left| {{x_2}} \right|^q} \leqslant s\left( {{x_1},{x_2}} \right)\frac{p}{{p+q}}{\left| {{x_1}} \right|^{p+q}}+{s^{ - p/q}}\left( {{x_1},{x_2}} \right)\frac{q}{{p+q}}{\left| {{x_2}} \right|^{p+q}}$$

### Lemma 3

^31^. Consider a nonlinear system$\dot {z}=f\left( {z,u} \right)$. If *V*(*z*) ≥ 0 be a smooth, continuous function and satisfying:

7 $$\dot {V}\left( z \right) \leqslant - {\rm H}{V^\gamma }\left( z \right)+N,$$

where *H* > 0, 0 < *γ* < 1 and 0 < *N* < ∞. Then, the nonlinear system $\dot {z}=f\left( {z,u} \right)$ is practical finite-time stable (PFTS), and the reach time T holds

8 $$T \leqslant {T_c}=\hbox{max} \frac{1}{{(1 - \gamma )v{\rm H}}}\left[ {{V^{1 - \gamma }}(z(0)) - {{\left( {\frac{N}{{(1 - v){\rm H}}}} \right)}^{(1 - \gamma )/\gamma }}} \right],$$

where *0 < v < 1.*

## Controller algorithm

**Fig. 2:**
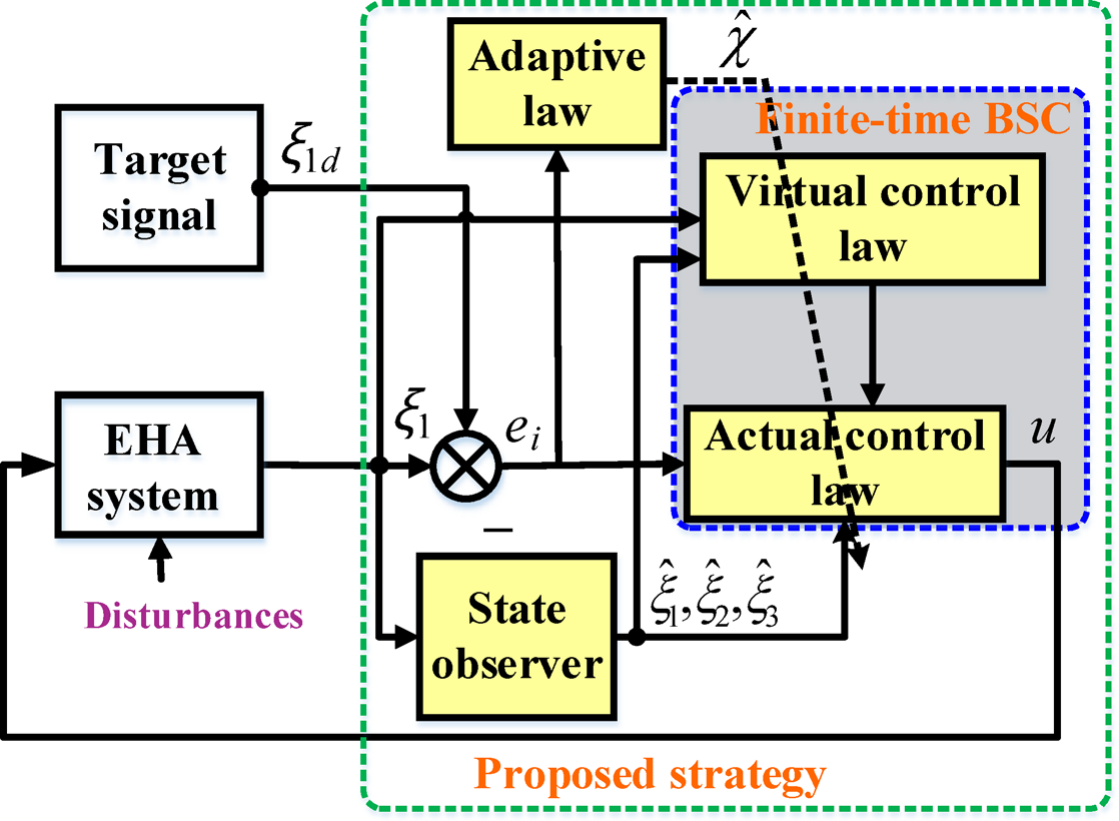
Topology of the proposed strategy.

In this section, the integration of state estimation into the finite-time backstepping approach and adaptive control law is investigated to design a robust controller and maintain precise tracking control. The structure of the proposed methodology is illustrated in Fig. 2. Herein, a state observer (SOB) is developed to estimate unmeasured states, while an adaptive finite-time backstepping controller is proposed to enhance tracking performance. The components shown in Fig. 2 are provided as follows:

### State observer (SOB)

The state observer can be established as follows^30^:

9 $$\left\{ {\begin{array}{*{20}{l}} {{{\dot {\hat {\xi }}}_1}={{\hat {\xi }}_2}+{\alpha _1}\left( {{{\tilde {\xi }}_1}+si{g^\beta }\left( {{{\tilde {\xi }}_1}} \right)} \right)} \\ {{{\dot {\hat {\xi }}}_2}={{\hat {\xi }}_3}+{\alpha _2}\left( {{{\tilde {\xi }}_1}+si{g^\beta }\left( {{{\tilde {\xi }}_1}} \right)} \right)} \\ {{{\dot {\hat {\xi }}}_3}=fu+{\alpha _3}\left( {{{\tilde {\xi }}_1}+si{g^\beta }\left( {{{\tilde {\xi }}_1}} \right)} \right)} \end{array}} \right.$$

where ${\tilde {\xi }_i}={\xi _i} - {\hat {\xi }_i},i=1,2,3$ denotes the estimation state errors, and $si{g^\beta }\left( {{{\tilde {\xi }}_1}} \right)={\left| {sign\left( {{{\tilde {\xi }}_1}} \right)} \right|^\beta }sign\left( {{{\tilde {\xi }}_1}} \right)$.

From (4) and (9), the dynamics of the system state error are calculated as follows^30^:

10 $$\dot {\tilde {\xi }}=P\tilde {\xi }+Q+D - Csi{g^\beta }\left( {{{\tilde {\xi }}_1}} \right)$$

where *P*, *Q*, and *D* are system matrices that are described as:

$$P=\left[ {\begin{array}{*{20}{c}} { - {\alpha _1}}&1&0 \\ { - {\alpha _2}}&0&1 \\ { - {\alpha _3}}&0&0 \end{array}} \right],Q=\left[ {\begin{array}{*{20}{c}} 0 \\ {{q_1}{\xi _2}} \\ {{q_2}{\xi _2}+{q_3}{\xi _3}} \end{array}} \right],D=\left[ {\begin{array}{*{20}{c}} 0 \\ {{d_1}} \\ {{d_2}} \end{array}} \right],C=\left[ {\begin{array}{*{20}{c}} {{\alpha _1}} \\ {{\alpha _2}} \\ {{\alpha _3}} \end{array}} \right]$$

where *α*_*i*_ (*i* = 1,2,3) are chosen to the matrix *P* is Hurwitz.

#### Remark 2

There is existing positive determinate matrix *F* such that the equation below is satisfied: *P*^*T*^*F* + *FP* = −*c*_1_*I*, where *I* is the unit matrix.

The Lyapunov function (LFU) can be chosen as follows:

11 $${V_s}={\tilde {\xi }^T}F\tilde {\xi }$$

The time derivative of *V*_*s*_ can be calculated by

12 $$\begin{gathered} {{\dot {V}}_s}={{\tilde {\xi }}^T}F\dot {\tilde {\xi }}+{{\dot {\tilde {\xi }}}^T}F\tilde {\xi } \hfill \\ ={{\tilde {\xi }}^T}\left( {{P^T}F+FP} \right)\dot {\tilde {\xi }}+2{{\tilde {\xi }}^T}FQ+2{{\tilde {\xi }}^T}FD - 2{{\tilde {\xi }}^T}FCsi{g^\beta }\left( {\tilde {\xi }} \right) \hfill \\ \end{gathered}$$

#### Lemma 4

Considering the nonlinear function *h*, the RBF neural network is used to approximate *h*, for instance, *h*(*s*) = *ω*^*T*^*R*(s) + *σ*(s). In detail, *R*(s) = [*R*_1_(s), *R*_2_(s), …, *R*_*m*_(s)]^T^ defines gaussian function that satisfies ||*R*|| ≤ 1. *σ*(s) is an NN approximation error, |*σ*(s)| < *σ*_*m*_(s) and *σ*_*m*_(s) > 0,*ω*^*T*^ = [*ω*_1_, …, *ω*_*m*_] represents an ideal NN weight vector. The expression can be written as follows:

13 $$\begin{gathered} \sigma =\mathop {\arg \hbox{min} }\limits_{{\omega \in {{\mathbb{R}}^N}}} \left\{ {\mathop {\sup \left| {g\left( s \right) - {{\hat {\omega }}^T}R\left( s \right)} \right|}\limits_{{s \in \Omega }} } \right\} \hfill \\ {\text{and }}{\left\| {{{\tilde {\omega }}_i}} \right\|^2}={\left\| {{{\hat {\omega }}_i} - {\omega _i}} \right\|^2} \leqslant {\chi _i},i=0,1,2,3 \hfill \\ \end{gathered}$$

where$\hat {\chi },\chi ,{\text{ and }}\tilde {\chi }=\hat {\chi } - \chi$ define the estimation, unknown actual value, and estimation error $\chi =\mathop {m{\mathrm{ax}}}\limits_{{i=0,1,2,3}} \left\{ {{\chi _i}} \right\},{\chi _i}={\left\| {{\omega _i}} \right\|^2}$.

Using RBF neural networks to approximate the continuous function *Q*, we have

14 $$\begin{gathered} \left\| Q \right\|={g_0}\left( s \right)=\omega _{0}^{T}{R_0}+{\sigma _0} \hfill \\ {\text{and }}{\left\| Q \right\|^2} \leqslant 2{\left\| {{\omega _0}} \right\|^2}{\left\| {{R_0}} \right\|^2}+2{\left\| {{\sigma _0}} \right\|^2} \leqslant 2{\chi _0}+2{\sigma _{0m}} \leqslant 2\chi +2{\sigma _m} \hfill \\ \end{gathered}$$

where $${\left\| {{\sigma _i}} \right\|^2} \leqslant {\sigma _{im}},i=0,1,2,3$$.

Applying the Young’s inequality, it yields

15 $$\begin{gathered} 2{{\tilde {\xi }}^T}FQ \leqslant {\varepsilon _1}{\left\| {\tilde {\xi }} \right\|^2}+\frac{1}{{{\varepsilon _1}}}{\left\| F \right\|^2}{\left\| Q \right\|^2} \leqslant {\varepsilon _1}{\left\| {\tilde {\xi }} \right\|^2}+\frac{{{{\left\| F \right\|}^2}}}{{{\varepsilon _1}}}\left( {2\chi +2{\sigma _m}} \right) \hfill \\ 2{{\tilde {\xi }}^T}FD \leqslant {\varepsilon _2}{\left\| {\tilde {\xi }} \right\|^2}+\frac{{{{\left\| F \right\|}^2}{D_m}}}{{{\varepsilon _2}}},{D_m}=\sum\limits_{{i=1}}^{2} {D_{i}^{2}} \hfill \\ - 2{{\tilde {\xi }}^T}FCsi{g^\beta }\left( {\tilde {\xi }} \right) \leqslant {\varepsilon _3}{\left\| {\tilde {\xi }} \right\|^2}+\frac{{{{\left\| {FC} \right\|}^2}}}{{{\varepsilon _3}}} \hfill \\ \end{gathered}$$

By substituting Eq. (15) into Eq. (12), one obtains

16 $${\dot {V}_s} \leqslant - \Gamma {\left\| {\tilde {\xi }} \right\|^2}+{\kappa _0}$$

where $$\Gamma =\left( {{c_1}I - {\varepsilon _1} - {\varepsilon _2} - {\varepsilon _3}} \right)$$and$${\kappa _0}=\frac{{{{\left\| F \right\|}^2}}}{{{\varepsilon _1}}}\left( {2\chi +2{\sigma _m}} \right)+\frac{{{{\left\| F \right\|}^2}{D_m}}}{{{\varepsilon _2}}}+\frac{{{{\left\| {FC} \right\|}^2}}}{{{\varepsilon _3}}}$$.

### Synthesis controller

Step 1: The tracking errors are calculated as follows:

17 $$\begin{gathered} {e_1}={{\hat {\xi }}_1} - {\xi _{1d}} \hfill \\ {e_i}={{\hat {\xi }}_i} - {\eta _{i - 1}},i=2,3 \hfill \\ \end{gathered}$$

where *ξ*_1*d*_ denotes the desired signal, *η*_*i*_ are the intermediate factors.

Taking derivative of (17), yields:

18 $${\dot {e}_1}={\hat {\xi }_2}+{\alpha _1}{\tilde {\xi }_1} - {\dot {\xi }_{1d}}={e_2}+{\eta _1}+{\alpha _1}{\tilde {\xi }_1} - {\dot {\xi }_{1d}}$$

We propose the virtual control law as follows:

19 $${\eta _1}= - {\lambda _1}e_{1}^{{2\gamma - 1}} - \frac{1}{{2{b_1}}}\hat {\chi }{e_1}+{\dot {\xi }_{1d}}$$

where *λ*_1_ and *b*_1_ are positive constant, γ = (2*a*_0_-1)/(2*a*_0_+1), *a*_0_ > 1.

The Lyapunov function candidate (LFC) can be formulated as follows:

20 $${V_1}={V_s}+\frac{1}{2}e_{1}^{2}+\frac{1}{{2{\lambda _0}}}{\tilde {\chi }^2}$$

Taking derivative of (20), yields:

21 $$\begin{gathered} {{\dot {V}}_1}={{\dot {V}}_s}+{e_1}{{\dot {e}}_1}+\frac{1}{{{\lambda _0}}}\tilde {\chi }\dot {\tilde {\chi }}={{\dot {V}}_s}+{e_1}\left( {{e_2}+{\eta _1}+{\alpha _1}{{\tilde {\xi }}_1} - {{\dot {\xi }}_{1d}}} \right)+\frac{1}{{{\lambda _0}}}\tilde {\chi }\dot {\hat {\chi }} \hfill \\ ={{\dot {V}}_s}+{e_1}{e_2} - {\lambda _1}e_{1}^{{2\gamma }} - \frac{1}{{2{b_1}}}\hat {\chi }e_{1}^{2}+{\alpha _1}{e_1}{{\tilde {\xi }}_1}+\frac{1}{{{\lambda _0}}}\tilde {\chi }\dot {\hat {\chi }} \hfill \\ \end{gathered}$$

From Lemma 1, one obtains:

22 $$\begin{gathered} {e_1}{e_2} \leqslant \frac{1}{{2{k_{11}}}}e_{2}^{2}+\frac{{{k_{11}}}}{2}e_{1}^{2} \hfill \\ {\alpha _1}{e_1}{{\tilde {\xi }}_1} \leqslant \frac{1}{{2{k_{12}}}}\tilde {\xi }_{1}^{2}+\frac{{{k_{12}}}}{2}\alpha _{1}^{2}e_{1}^{2} \hfill \\ \end{gathered}$$

Defines $${\Psi _1}=\left( {\frac{{{k_{11}}}}{2}+\frac{{{k_{12}}}}{2}\alpha _{1}^{2}} \right){e_1}=\omega _{1}^{T}{R_1}+{\sigma _1}$$.

Applying the Young’s inequality with noted that $\chi =\hbox{max} \left( {{\chi _i}} \right)$, we have

23 $${e_1}{\Psi _1}={e_1}\omega _{1}^{T}{R_1}+{e_1}{\sigma _1} \leqslant \frac{1}{{2{b_1}}}\chi e_{1}^{2}+\frac{{{b_1}}}{2}+\frac{1}{{2\gamma }}e_{1}^{{2\gamma }}+\left( {1 - \frac{1}{{2\gamma }}} \right)\sigma _{{1m}}^{{\frac{{2\gamma }}{{2\gamma - 1}}}}$$

By substituting (23) into Eq. (21), leads to

24 $$\begin{gathered} {{\dot {V}}_1} \leqslant - \Gamma {\left\| {\tilde {\xi }} \right\|^2}+{\kappa _0}+\frac{1}{{2{k_{11}}}}e_{2}^{2}+\frac{1}{{2{k_{12}}}}\tilde {\xi }_{1}^{2} - \frac{1}{{2{b_1}}}\tilde {\chi }e_{1}^{2}+\frac{{{b_1}}}{2}+\frac{1}{{2\gamma }}e_{1}^{{2\gamma }}+\left( {1 - \frac{1}{{2\gamma }}} \right)\sigma _{{1m}}^{{\frac{{2\gamma }}{{2\gamma - 1}}}} \hfill \\ - {\lambda _1}e_{1}^{{2\gamma }}+\frac{1}{{{\lambda _0}}}\tilde {\chi }\dot {\hat {\chi }} \hfill \\ {{\dot {V}}_1} \leqslant - \left( {\Gamma - \frac{1}{{2{k_{12}}}}} \right){\left\| {\tilde {\xi }} \right\|^2} - \left( {{\lambda _1} - \frac{1}{{2\gamma }}} \right)e_{1}^{{2\gamma }}+{\kappa _0}+\frac{{{b_1}}}{2}+\left( {1 - \frac{1}{{2\gamma }}} \right)\sigma _{{1m}}^{{\frac{{2\gamma }}{{2\gamma - 1}}}}+\frac{1}{{2{k_{11}}}}e_{2}^{2} \hfill \\ +\tilde {\chi }\left( {\frac{1}{{{\lambda _0}}}\dot {\hat {\chi }} - \frac{1}{{2{b_1}}}e_{1}^{2}} \right) \hfill \\ {{\dot {V}}_1} \leqslant - \left( {\Gamma - \frac{1}{{2{k_{12}}}}} \right){\left\| {\tilde {\xi }} \right\|^2} - \left( {{\lambda _1} - \frac{1}{{2\gamma }}} \right)e_{1}^{{2\gamma }}+\frac{1}{{2{k_{11}}}}e_{2}^{2}+\tilde {\chi }\left( {\frac{1}{{{\lambda _0}}}\dot {\hat {\chi }} - \frac{1}{{2{b_1}}}e_{1}^{2}} \right)+{\kappa _1} \hfill \\ \end{gathered}$$

where $${\kappa _1}={\kappa _0}+\frac{{{b_1}}}{2}+\left( {1 - \frac{1}{{2\gamma }}} \right)\sigma _{{1m}}^{{\frac{{2\gamma }}{{2\gamma - 1}}}}$$.

Step 2:

Taking derivative the error between virtual signal *η*_1_ and *ξ*_2_ in (17) with respect to time, one yields

25 $${\dot {e}_2}={\hat {\xi }_3}+{\alpha _2}{\tilde {\xi }_1} - {\dot {\eta }_1}={e_3}+{\eta _2}+{\alpha _2}{\tilde {\xi }_1} - {\dot {\eta }_1}$$

We propose the virtual control law as follows:

26 $${\eta _2}= - {\lambda _2}e_{2}^{{2\gamma - 1}} - \frac{1}{{2{b_2}}}\hat {\chi }{e_2}+{\dot {\eta }_1}$$

where *λ*_2_ and *b*_2_ are positive gains.

The Lyapunov function in this step can be given as follows:

27 $${V_2}={V_1}+\frac{1}{2}e_{2}^{2}$$

Differentiating of (27), one obtains:

28 $$\begin{gathered} {{\dot {V}}_1}={{\dot {V}}_1}+{e_2}{{\dot {e}}_2}={{\dot {V}}_1}+{e_2}\left( {{e_3}+{\eta _2}+{\alpha _2}{{\tilde {\xi }}_1} - {{\dot {\eta }}_1}} \right) \hfill \\ ={{\dot {V}}_1}+{e_2}{e_3} - {\lambda _2}e_{2}^{{2\gamma }} - \frac{1}{{2{b_2}}}\hat {\chi }e_{2}^{2}+{\alpha _2}{e_2}{{\tilde {\xi }}_1} \hfill \\ \end{gathered}$$

From Lemma 1, one obtains:

29 $$\begin{gathered} {e_2}{e_3} \leqslant \frac{1}{{2{k_{21}}}}e_{3}^{2}+\frac{{{k_{21}}}}{2}e_{2}^{2} \hfill \\ {\alpha _2}{e_2}{{\tilde {\xi }}_1} \leqslant \frac{1}{{2{k_{22}}}}\tilde {\xi }_{1}^{2}+\frac{{{k_{22}}}}{2}\alpha _{2}^{2}e_{2}^{2} \hfill \\ \end{gathered}$$

Defines $${\Psi _2}=\left( {\frac{{{k_{21}}}}{2}+\frac{{{k_{22}}}}{2}\alpha _{2}^{2}+\frac{1}{{2{k_{11}}}}} \right){e_2}=\omega _{2}^{T}{R_2}+{\sigma _2}$$.

Applying the Young’s inequality with noted that $\chi =\hbox{max} \left( {{\chi _i}} \right)$, we have

30 $${e_2}{\Psi _2}={e_2}\omega _{2}^{T}{R_2}+{e_2}{\sigma _2} \leqslant \frac{1}{{2{b_2}}}\chi e_{2}^{2}+\frac{{{b_2}}}{2}+\frac{1}{{2\gamma }}e_{2}^{{2\gamma }}+\left( {1 - \frac{1}{{2\gamma }}} \right)\sigma _{{2m}}^{{\frac{{2\gamma }}{{2\gamma - 1}}}}$$

By substituting (30) and (29) into Eq. (28), leads to

31 $$\begin{gathered} {{\dot {V}}_2} \leqslant {{\dot {V}}_1}+\frac{1}{{2{k_{21}}}}e_{3}^{2}+\frac{1}{{2{k_{22}}}}\tilde {\xi }_{1}^{2}+\frac{1}{{2{b_2}}}\chi e_{2}^{2}+\frac{{{b_2}}}{2}+\frac{1}{{2\gamma }}e_{2}^{{2\gamma }}+\left( {1 - \frac{1}{{2\gamma }}} \right)\sigma _{{2m}}^{{\frac{{2\gamma }}{{2\gamma - 1}}}} - {\lambda _2}e_{2}^{{2\gamma }} - \frac{1}{{2{b_2}}}\hat {\chi }e_{2}^{2} \hfill \\ {{\dot {V}}_2} \leqslant - \left( {\Gamma - \frac{1}{{2{k_{12}}}} - \frac{1}{{2{k_{22}}}}} \right){\left\| {\tilde {\xi }} \right\|^2} - \sum\limits_{{i=1}}^{2} {\left( {{\lambda _i} - \frac{1}{{2\gamma }}} \right)e_{i}^{{2\gamma }}} +\frac{1}{{2{k_{21}}}}e_{3}^{2}+\tilde {\chi }\left( {\frac{1}{{{\lambda _0}}}\dot {\hat {\chi }} - \sum\limits_{{i=1}}^{2} {\frac{1}{{2{b_i}}}e_{i}^{2}} } \right)+{\kappa _2} \hfill \\ \end{gathered}$$

where $${\kappa _2}={\kappa _1}+\frac{{{b_2}}}{2}+\left( {1 - \frac{1}{{2\gamma }}} \right)\sigma _{{2m}}^{{\frac{{2\gamma }}{{2\gamma - 1}}}}$$.

Step 3:

Taking derivative the error between virtual signal *η*_2_ and *ξ*_3_ in (17) with respect to time, one yields

32 $${\dot {e}_3}={\dot {\hat {\xi }}_3} - {\dot {\eta }_2}=fu+{\alpha _3}{\tilde {\xi }_1} - {\dot {\eta }_2}$$

The virtual control law can be suggested by:

33 $$u=\frac{1}{f}\left( { - {\lambda _3}e_{3}^{{2\gamma - 1}} - \frac{1}{{2{b_3}}}\hat {\chi }{e_3}+{{\dot {\eta }}_2}} \right)$$

where *λ*_3_ and *b*_3_ are positive gains.

The Lyapunov function in this step can be selected as follows:

34 $${V_3}={V_2}+\frac{1}{2}e_{3}^{2}$$

Differentiating of (34), one yields:

35 $$\begin{aligned} \dot{V}_{3} = & \dot{V}_{2} + e_{3} \dot{e}_{3} = \dot{V}_{2} + e_{3} \left( {fu + \alpha _{3} \tilde{\xi }_{1} - \dot{\eta }_{2} } \right) \\ & = \dot{V}_{{23}} - \lambda _{3} e_{3}^{{2\gamma }} - \frac{1}{{2b_{3} }}\hat{\chi }e_{3}^{2} + \alpha _{3} e_{3} \tilde{\xi }_{1} \\ \end{aligned}$$

From Lemma 1, one obtains:

36 $${\alpha _3}{e_3}{\tilde {\xi }_1} \leqslant \frac{1}{{2{k_{32}}}}\tilde {\xi }_{1}^{2}+\frac{{{k_{32}}}}{2}\alpha _{3}^{2}e_{3}^{2}$$

Defines $${\Psi _3}=\left( {\frac{{{k_{32}}}}{2}\alpha _{3}^{2}+\frac{1}{{2{k_{21}}}}} \right){e_3}=\omega _{3}^{T}{R_3}+{\sigma _3}$$.

Applying the Young’s inequality with noted that $\chi =\hbox{max} \left( {{\chi _i}} \right)$, we have

37 $${e_3}{\Psi _3}={e_3}\omega _{3}^{T}{R_3}+{e_3}{\sigma _3} \leqslant \frac{1}{{2{b_3}}}\chi e_{3}^{2}+\frac{{{b_3}}}{2}+\frac{1}{{2\gamma }}e_{3}^{{2\gamma }}+\left( {1 - \frac{1}{{2\gamma }}} \right)\sigma _{{3m}}^{{\frac{{2\gamma }}{{2\gamma - 1}}}}$$

By substituting (36) and (37) into Eq. (35), leads to

38 $$\begin{gathered} {{\dot {V}}_3} \leqslant {{\dot {V}}_3}+\frac{1}{{2{k_{32}}}}\tilde {\xi }_{1}^{2}+\frac{1}{{2{b_3}}}\chi e_{3}^{2}+\frac{{{b_3}}}{2}+\frac{1}{{2\gamma }}e_{3}^{{2\gamma }}+\left( {1 - \frac{1}{{2\gamma }}} \right)\sigma _{{3m}}^{{\frac{{2\gamma }}{{2\gamma - 1}}}} - {\lambda _3}e_{3}^{{2\gamma }} - \frac{1}{{2{b_3}}}\hat {\chi }e_{3}^{2} \hfill \\ {{\dot {V}}_3} \leqslant - \left( {\Gamma - \frac{1}{{2{k_{12}}}} - \frac{1}{{2{k_{22}}}} - \frac{1}{{2{k_{32}}}}} \right){\left\| {\tilde {\xi }} \right\|^2} - \sum\limits_{{i=1}}^{3} {\left( {{\lambda _i} - \frac{1}{{2\gamma }}} \right)e_{i}^{{2\gamma }}} +\tilde {\chi }\left( {\frac{1}{{{\lambda _0}}}\dot {\hat {\chi }} - \sum\limits_{{i=1}}^{3} {\frac{1}{{2{b_i}}}e_{i}^{2}} } \right)+{\kappa _3} \hfill \\ \end{gathered}$$

where $${\kappa _3}={\kappa _2}+\frac{{{b_3}}}{2}+\left( {1 - \frac{1}{{2\gamma }}} \right)\sigma _{{3m}}^{{\frac{{2\gamma }}{{2\gamma - 1}}}}$$.

The adaptive law $\dot {\hat {\chi }}$ is established as follows:

39 $$\dot {\hat {\chi }}={\lambda _0}\left( {\sum\limits_{{i=1}}^{3} {\frac{1}{{2{b_i}}}e_{i}^{2}} - {\lambda _x}\hat {\chi }} \right)$$

where *λ*_*x*_ defines the positive constant.

#### Remark 3

In this paper, the NN adaptation law (Eq. (39)) is based on the “maximum norm” approach$\chi =\hbox{max} \left( {{\chi _i}} \right)$. This method decreases the number of parameters to be estimated. In addition, the constraints associated with conventional norm-based estimation inequalities are significantly relaxed, while preserving the essential properties of the original system.

By substituting (39) into (38), and rearranging yields:

40 $${\dot {V}_3} \leqslant - {\Gamma _m}{\tilde {\xi }^T}Q\tilde {\xi } - \sum\limits_{{i=1}}^{3} {{\lambda _{mi}}e_{i}^{{2\gamma }}} - {\lambda _x}\tilde {\chi }\hat {\chi }+{\kappa _3}$$

where $${\Gamma _m}={{\left( {\Gamma - \frac{1}{{2{k_{12}}}} - \frac{1}{{2{k_{22}}}} - \frac{1}{{2{k_{32}}}}} \right)} \mathord{\left/ {\vphantom {{\left( {\Gamma - \frac{1}{{2{k_{12}}}} - \frac{1}{{2{k_{22}}}} - \frac{1}{{2{k_{32}}}}} \right)} {\left\| {{\lambda _{\hbox{max} }}\left( Q \right)} \right\|}}} \right. \kern-0pt} {\left\| {{\lambda _{\hbox{max} }}\left( Q \right)} \right\|}},{\lambda _{mi}}={\lambda _i} - \frac{1}{{2\gamma }}$$.

Utilizing the Young’s inequality, one can yields

41 $$- {\lambda _x}\tilde {\chi }\hat {\chi } \leqslant \frac{{{\lambda _x}}}{2}{\chi ^2} - \frac{{{\lambda _x}}}{2}{\tilde {\chi }^2}$$

Hence, (40) leads to

42 $${\dot {V}_3} \leqslant - {\Gamma _m}{\tilde {\xi }^T}Q\tilde {\xi } - \sum\limits_{{i=1}}^{3} {{\lambda _{mi}}e_{i}^{{2\gamma }}} - \frac{{{\lambda _x}}}{2}{\tilde {\chi }^2}+{\kappa _3}+\frac{{{\lambda _x}}}{2}{\chi ^2}$$

From Lemma 2, we can get:

43 $$\begin{gathered} {\left( {{{\tilde {\xi }}^T}Q\tilde {\xi }} \right)^\gamma } \leqslant \left( {1 - \gamma } \right){\gamma ^{\frac{\gamma }{{1 - \gamma }}}}+{{\tilde {\xi }}^T}Q\tilde {\xi } \hfill \\ {\left( {\frac{1}{{2{\lambda _0}}}{{\tilde {\chi }}^2}} \right)^\gamma } \leqslant \left( {1 - \gamma } \right){\gamma ^{\frac{\gamma }{{1 - \gamma }}}}+\frac{1}{{2{\lambda _0}}}{{\tilde {\chi }}^2} \hfill \\ \end{gathered}$$

By substituting (43) into Eq. (42), leads to

44 $$\begin{gathered} {{\dot {V}}_3} \leqslant - {\Gamma _m}{\left( {{{\tilde {\xi }}^T}Q\tilde {\xi }} \right)^\gamma } - \sum\limits_{{i=1}}^{3} {{\lambda _{mi}}e_{i}^{{2\gamma }}} - {\lambda _{xm}}{\left( {\frac{1}{{2{\lambda _0}}}{{\tilde {\chi }}^2}} \right)^\gamma }+{\kappa _{3m}} \hfill \\ {{\dot {V}}_3} \leqslant - {\rm H}V_{3}^{\gamma }+{\rm N} \hfill \\ \end{gathered}$$

where $${\kappa _{3m}}={\kappa _3}+{\Gamma _m}\left( {1 - \gamma } \right){\gamma ^{\frac{\gamma }{{1 - \gamma }}}}+{\lambda _{xm}}\left( {1 - \gamma } \right){\gamma ^{\frac{\gamma }{{1 - \gamma }}}}+\frac{{{\lambda _x}}}{2}{\chi ^2},{\lambda _{xm}}={\lambda _x}{\lambda _0}$$.

#### Theorem 1

For the EPS system (4), under the presented SOB (9), the adaption law (39), the intermediate control law (19), (26) and actual controller (33), the error signals $\tilde {\xi },{e_i},\tilde {\chi }$ and system states of the EPS system are PFTS and arrives at small region in finite time.

**Proof of Theorem** **1**. From (44) and based on Lemma 3, with ∀t ≥ T, $V_{3}^{\gamma }\left( {\tilde {\xi },{e_i},\tilde {\chi }} \right) \leqslant {\rm N}/\left( {\left( {1 - v} \right)H} \right)$, it can be concluded that all the signals of presented system are SGPFS. □.

#### Remark 4

Among the tuned control parameters, several gain parameters significantly influence the system performance, including ${\lambda}_{1}$, ${\lambda}_{2}$, ${\lambda}_{3}$, the fractional-order gain $\gamma$, the adaptation parameters ${\lambda}_{0}$and ${\lambda}_{x}$, and the SOB gains ${\alpha}_{i}$. According to Theorem 1, by selecting sufficiently large values for ${\lambda}_{1}$, ${\lambda}_{2}$, and ${\lambda}_{3}$, an appropriate fractional-order gain $\gamma$, and sufficiently small values for ${\lambda}_{0}$and ${\lambda}_{x}$, the tracking error, approximation error, and adaptation error can be driven to a small neighborhood around the origin in the finite-time. In future work, optimal tuning methods for these gain parameters will be investigated to further enhance the performance of the proposed controller.

## Simulation studies

### Simulation setup

To validate enhanced performance of the exploited control algorithm, simulation trials are first performed in the EHSC using the MATLAB/Simulink environment (R2023b version). The sinusoidal signal considered as desired trajectory is presented by

45 $${\xi _{1d}}={\text{ }}8{\text{ }}+{\text{ }}8sin(0.8\pi t - \pi /2)\,\,\,\,\left( {{\mathrm{mm}}} \right)$$

The value parameters of the EHSC are conducted in Table 1 which based on data presented in^32^.

**Table 1:** EHSC parameters.

Parameter	Value (Unit)	Parameter	Value (Unit)
*M*	4.5 (kg)	*β*_*e*_	1.25 × 10^3^ (MPa)
*A*_*s*_	4 × 10^-4^ (m^2^)	*k*_*SV*_	3.2 × 10^-8^ (m^3^/s/V/Pa^-1/2^)
*P*_*s*_	16 (MPa)	*b*	450 (Ns/m)
*V*_*a*_	6 × 10^-5^ (m^3^)	*C*_*s*_	1.2 × 10^-11^ (m^5^/Ns)

The advantages of the proposed method are validated through comparison with other control strategies, based on both simulation and experimental trials, as detailed below:

The first control strategy is adaptive neural network-based finite-time backstepping controller (ANNBC) that is designed by^33^

46 $$\begin{gathered} {z_1}={\xi _1} - {\xi _{1d}},{\eta _1}= - {k_{11}}{z_1} - {k_{12}}z_{1}^{\gamma }+{{\dot {x}}_{1d}}, \hfill \\ {z_2}={\xi _2} - {\eta _1},{\eta _2}= - {q_1}{\xi _2} - {k_{21}}{z_2} - {k_{22}}z_{2}^{\gamma } - \hat {W}_{1}^{T}{\phi _1}+{{\dot {\eta }}_1}, \hfill \\ {z_3}={\xi _3} - {\eta _2},u=\frac{1}{g}\left( { - {q_2}{\xi _2} - {q_3}{\xi _3}+{{\dot {\eta }}_2} - {k_{31}}{z_3} - {k_{32}}z_{3}^{\gamma } - {z_2}} \right) \hfill \\ \end{gathered}$$

where *k*_1*l*_ and *k*_2*l*_ (*l* = 1, 2, 3) are positive constants.

The second control strategy is an NNPID that is designed by^34^

47 $$\begin{gathered} {z_1}={\xi _1} - {\xi _{1d}}, \hfill \\ {u_{NNPID}}=\left( {{k_p}+\Delta {k_p}} \right){z_1}_{\prime }+\left( {{k_i}+\Delta {k_i}} \right)\int\limits_{0}^{\tau } {{z_1}d\tau } +\left( {{k_d}+\Delta {k_d}} \right)\frac{{d{z_1}}}{{dt}} \hfill \\ \end{gathered}$$

where *k*_*l*_ (*l* = *p*, *i*, *d*) are positive constants, Δ*k*_*l*_ = $\hat {W}_{l}^{T}{\phi _l}$ (*l* = *p*, *i*, *d*) are the output of NN.

For the proposed controller, the parameters for the SOB are set as *α*_1_ *=* 0.05; *α*_2_ *=* 0.4; *α*_3_ *=* 13; *β* = 97/99. The control gains are chosen as: *λ*_1_ *=* 70; *λ*_2_ *=* 130; *λ*_3_ *=* 80; *γ =* 7/9. The parameters for adaptation law are selected as *b*_1_ = *b*_2_ = *b*_3_ = 3; *λ*_0_ *=* 0.02; *λ*_*x*_ *=* 0.8. The external disturbance is simulated by *d*_1_ = 10*ξ*_2_ + 10tanh(*ξ*_2_), *d*_2_ = 6sin(πt) in the simulation.

For comparison, three performance indicators are given to confirm the usefulness of the suggested control method which can be presented as follows^35,36^:

48 $$\begin{gathered} {\mathrm{STD}}=\sqrt {\frac{1}{L}\sum\limits_{{i=1}}^{L} {\left| {{\xi _1}(i) - {\xi _{1d}}(i) - {\mathrm{AVE}}} \right|} } \hfill \\ {\mathrm{AVE}}=\frac{1}{L}\sum\limits_{{i=1}}^{L} {\left| {{\xi _1}(i) - {\xi _{1d}}(i)} \right|} \hfill \\ {\mathrm{MAXE}}=\mathop {\hbox{max} }\limits_{{i=1, \ldots ,L}} \left\{ {\left| {{\xi _1}(i) - {\xi _{1d}}(i)} \right|} \right\} \hfill \\ \end{gathered}$$

where *L* and ${\mathrm{AVE}}$denote the quantity of the sampled signals and the average error, respectively.

**Fig. 3:**
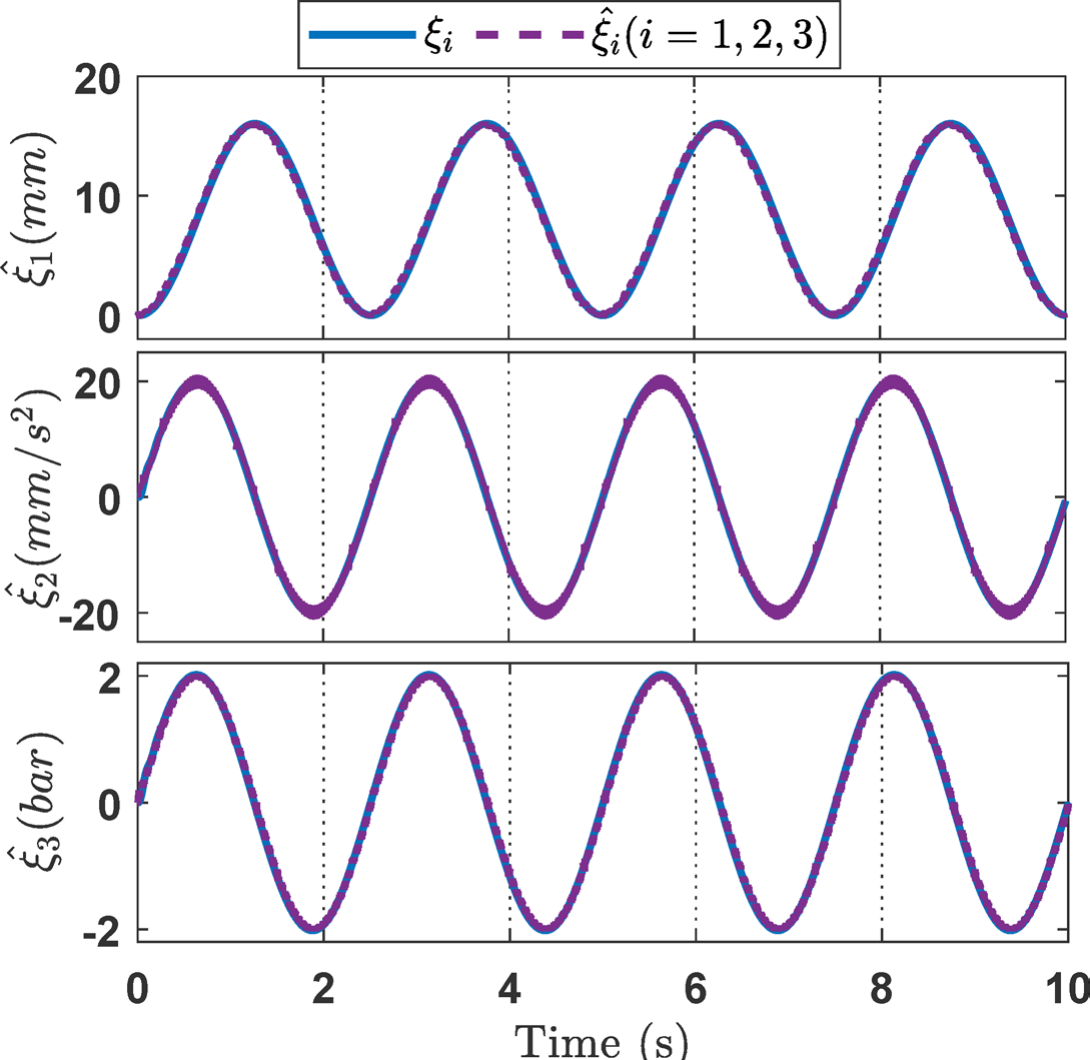
The estimation of state variables *ξ*_*i*_ (*i* = 1, 2, 3) in the simulation.

### Simulation result

**Fig. 4:**
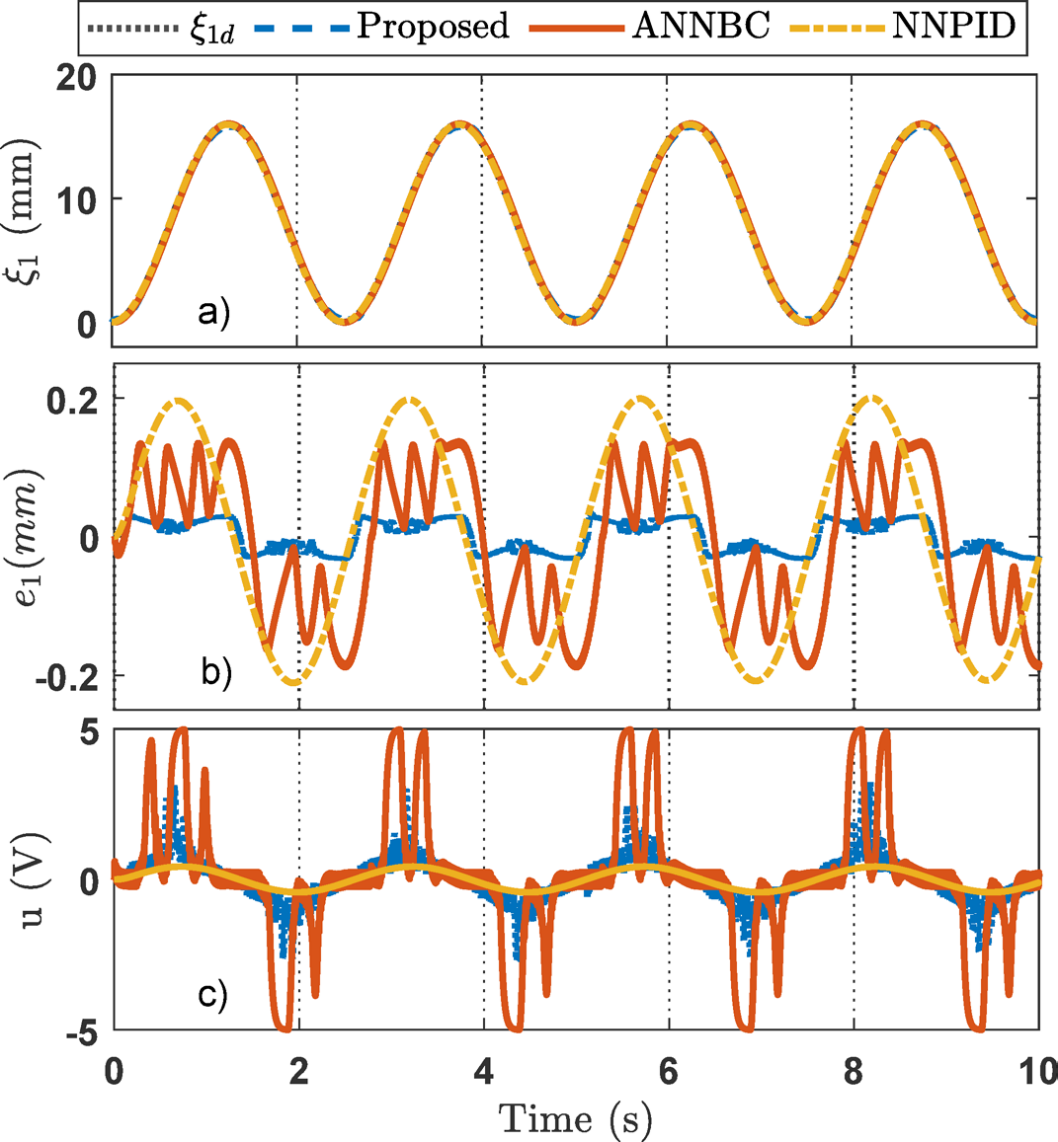
Output tracking control in the simulation: (**a**) tracking performance, (**b**) error, (**c**) control signal.

**Fig. 5:**
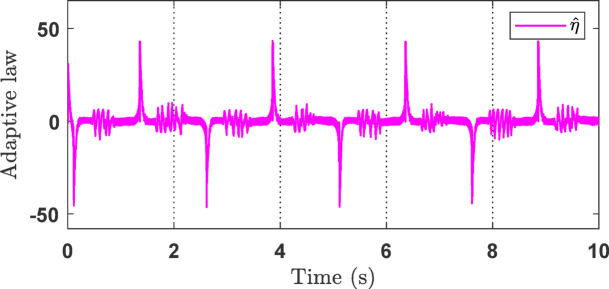
The *χ* adaptation of the proposed method in the simulation.

For the simulation studies, in the case where the desired trajectory is a sinusoidal signal as described in Eq. (45), the simulation results including variable state estimation, output tracking performance, and the adaptive law are presented in Figs. 3, 4 and 5. The total simulation time is 10s. The effectiveness of SOB estimation is illustrated in Fig. 3. It can be observed that the estimated values of the system state variables *ξ*_*i*_ (*i* = 1, 2, 3) satisfactorily track the actual values. The estimated state variables are fed into the control design process, which helps reduce hardware costs, such as those associated with pressure transducers and velocity sensors. Figure 4 shows the output response of the EHSC system, including displacement tracking, the deviation between desired and actual values, and the control action signal.

From Fig. 4a, it can be seen that ANNBC and proposed controllers provide acceptable tracking performance, with the actual values closely following the reference signal. Meanwhile, the NNPID controller exhibits poor performance, with a maximum tracking error of up to 0.2511 mm. As shown in Fig. 4b, the tracking error of the NNPID controller is the largest among the three methods. The ANNBC controller reduces the error compared to NNPID, but its performance is still inferior to that of the proposed control algorithm. Therefore, the proposed controller demonstrates the highest efficiency among the three approaches. This improved performance is attributed to the incorporation of finite-time backstepping and adaptive law techniques.

Figure 4c shows the control signals of the three controllers. It can be observed that the proposed controller produces high-frequency chattering due to the strong control effort required to compensate for disturbances and other adverse factors. Reducing this chattering effect will be addressed in future work. Figure 5 illustrates the capability of the adaptive law to handle unknown system dynamics, thereby supporting the performance of the main controller during operation.

**Fig. 6:**
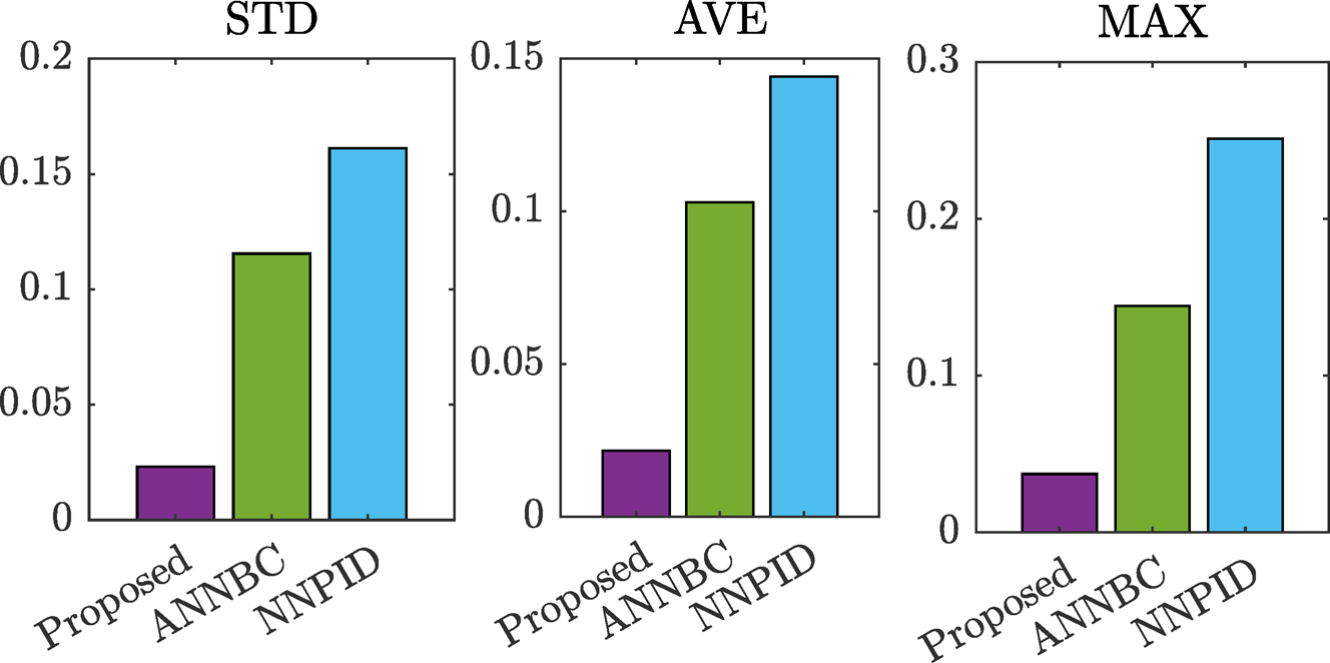
Comparison of indexes in the simulation.

Figure 6 summarizes the simulation performance indicators: standard deviation (STD), average error (AVE), and maximum error (MAX) for the three control strategies. For the proposed method, ANNBC, and NNPID, the STD values are 0.0231, 0.1155, and 0.1612, respectively. The AVE values are 0.0217, 0.1030, and 0.1442, respectively. Similarly, the MAX errors are 0.0373 for the proposed method, 0.1444 for ANNBC, and 0.2511 for NNPID. The reported data indicates that the proposed control strategy offers significant improvements compared to other approaches.

## Experimental validation results

### Experiment setup

**Fig. 7:**
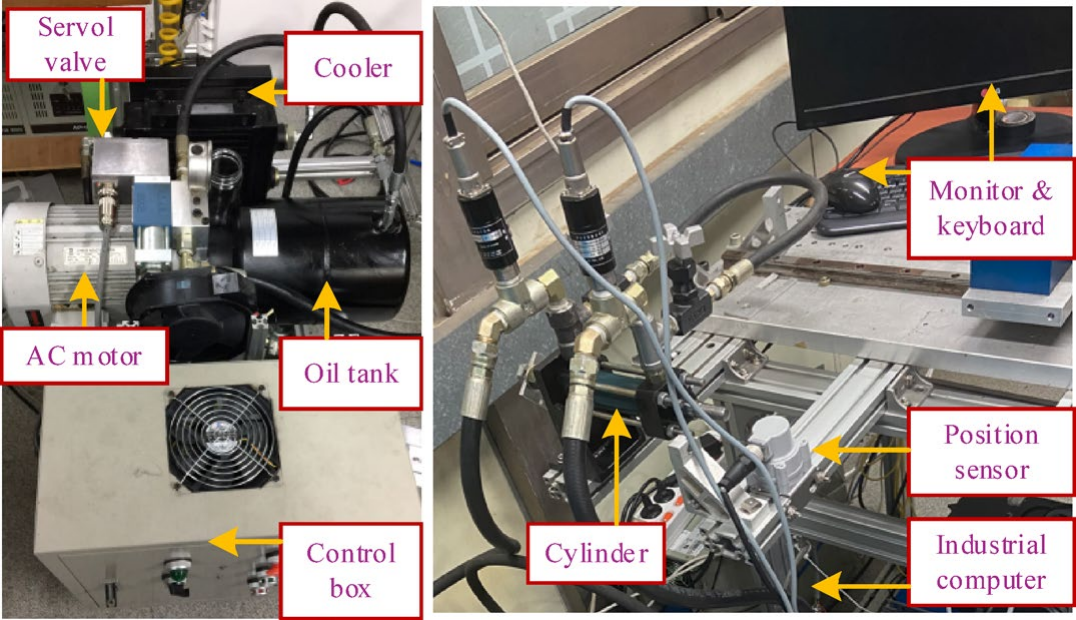
Experimental platform of the EHSC system.

The principal components of the EHSC system are shown in Fig. 7. In this system, the servo valve (Model D633-317B) is manufactured by Moog Company, and the hydraulic supply system includes a three-phase AC motor, an oil tank, a cooling system, and a relief valve. For the measurement system, only a position sensor (Rational WTB5-0500MM) is used, which is attached to the stroke of the symmetrical cylinder (35 mm). Regarding the software, MATLAB/SIMULINK is integrated and executed using an industrial computer connected to an NI PCI-6014 card. The developed control algorithm is embedded in MATLAB, and the resulting outputs are observed on the monitor.

In the experiment, two cases are performed to exam the advantages of the proposed method. First, the ramp signal is considered as the desired orbit that starts at a specified time (1s) and changes at a specified rate of 1 with the simulation time of 10s. Second, the target signal in experiment is selected as${\xi _{1d}}={\text{ }}10{\text{ }}+{\text{ }}10sin(2\pi t - \pi /2)\,\,\,\,\left( {{\mathrm{mm}}} \right)$ .

### Experiment results

#### Case 1

**Fig. 8:**
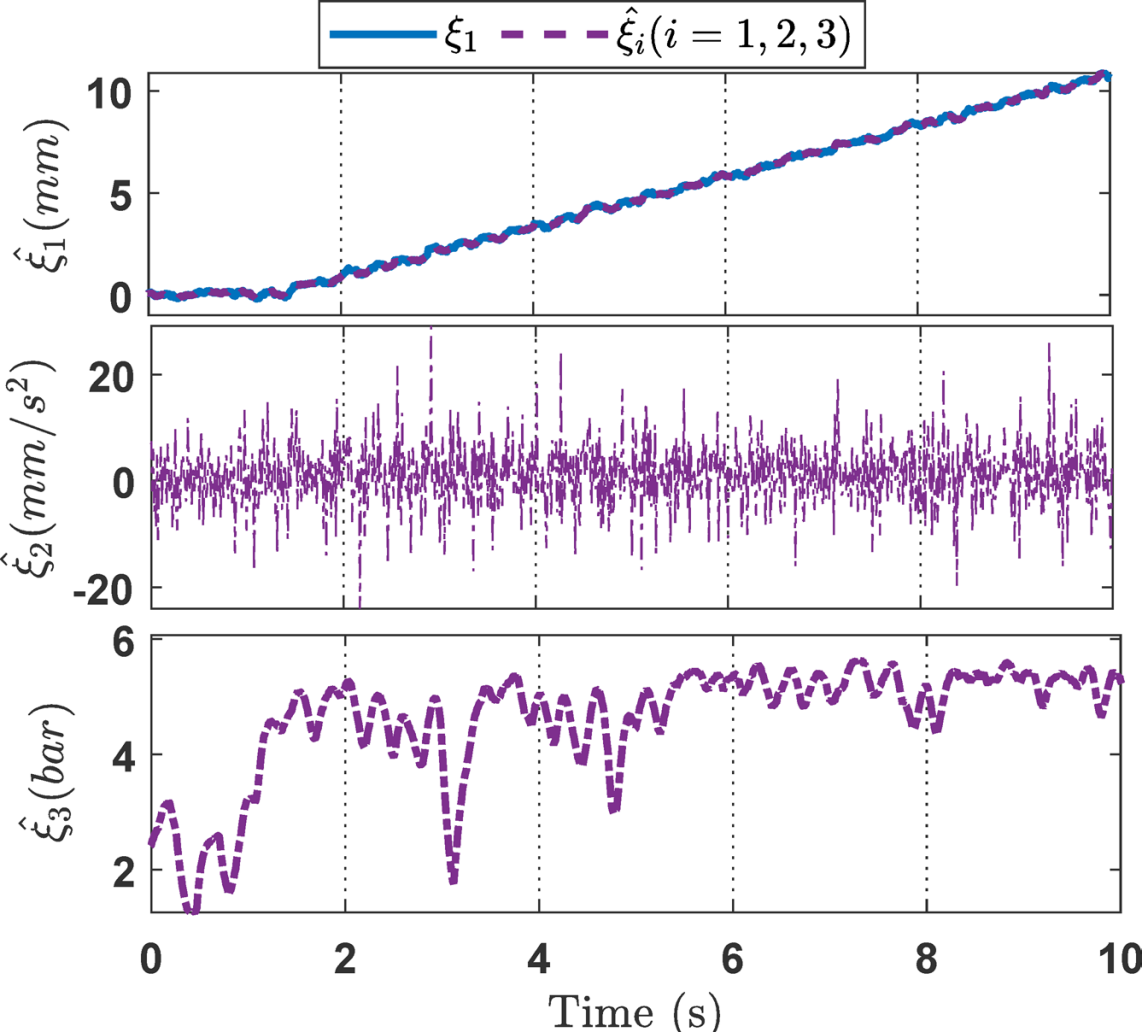
The estimation of state variables *ξ*_*i*_ (*i* = 1, 2, 3) in the ramp experiment.

**Fig. 9:**
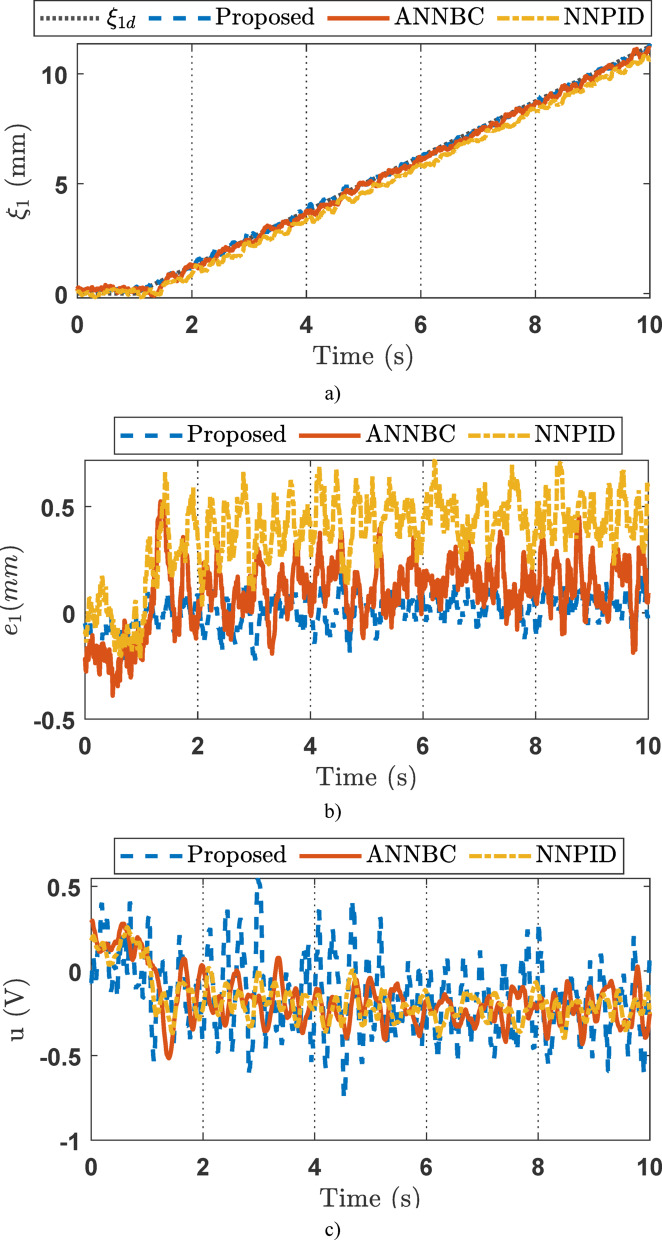
Output tracking control in the ramp experiment: (a) tracking performance, (b) error, (c) control signal.

**Fig. 10:**
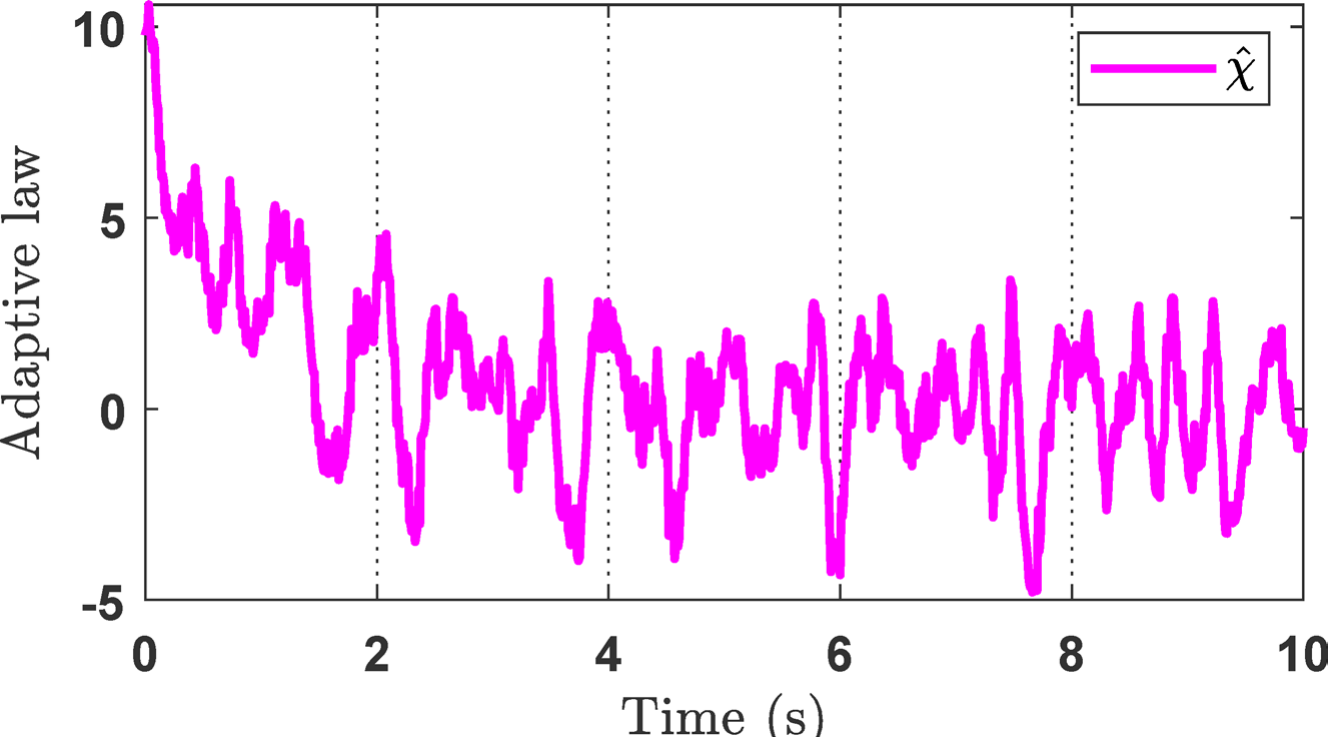
The *χ* adaptation of the proposed controller in the ramp experiment.

For the ramp signal experiment studies, the experiment results including variable state estimation, output tracking performance, and the adaptive law are presented in Figs. 8, 9 and 10. The total experiment time is 10s. The effectiveness of SOB estimation is exhibited on Fig. 8. It implies that the presented SOB can accurately estimate the unmeasurable states *ξ*_*i*_ (*i* = 1, 2, 3). Furthermore, incorporating the estimated state variables into the control design eliminates the requirement for full sensor instrumentation. Figure 9 presents the output tracking performance of the EHSC system. As shown in Fig. 9a, all three controllers achieve acceptable tracking performance, with actual outputs closely following the reference signal. However, Fig. 9b indicates that the NNPID controller exhibits the largest tracking error. While the ANNBC controller improves upon this, its performance remains inferior to that of the proposed controller. Hence it demonstrates that the proposed method outperforms both the NNPID and ANNBC controllers. This superior performance is attributed to the integration of finite-time backstepping and the adaptive law mechanism. The control signals shown in Fig. 9c indicate that the proposed controller requires a significantly higher control effort compared to the other methods. This is mainly due to the stronger control action needed to suppress disturbances and to respond to rapid changes in the desired signal. Finally, Fig. 10 illustrates the capability of the adaptive law to handle unknown system dynamics, thereby improving the robustness of the overall control strategy.

#### Case 2

**Fig. 11:**
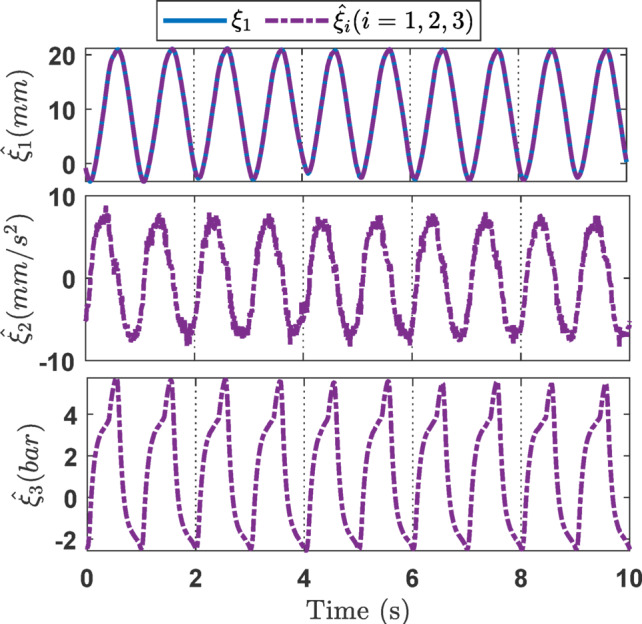
The estimation of state variables *ξ*_*i*_ (*i* = 1, 2, 3) in the sinusoidal experiment.

**Fig. 12:**
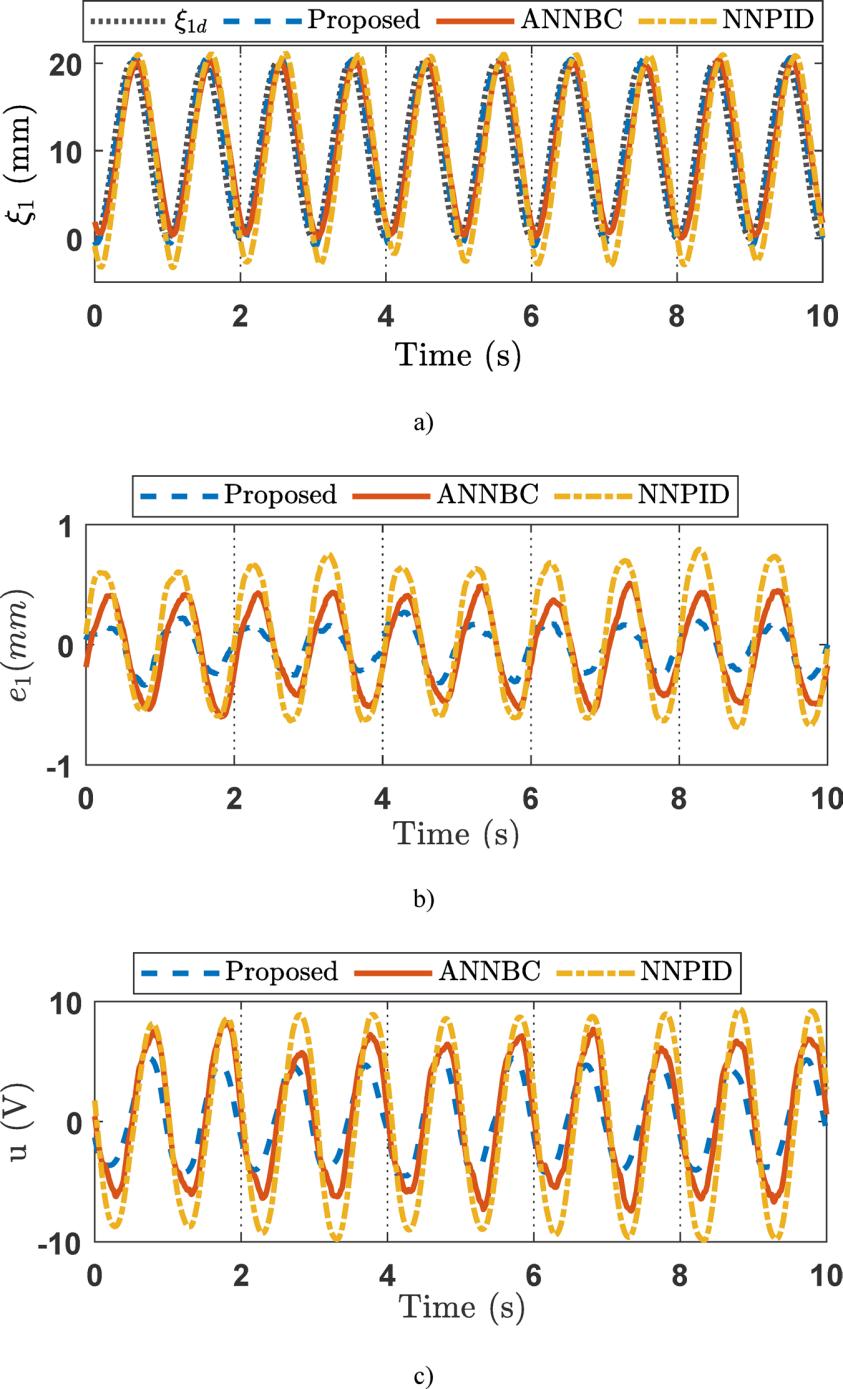
Output tracking control in the sinusoidal experiment: (a) tracking performance, (b) error, (c) control signal.

**Fig. 13:**
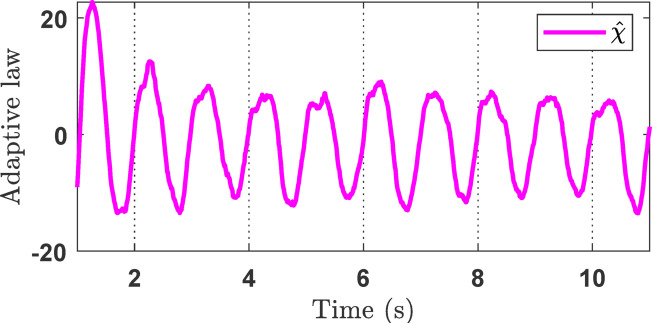
The *χ* adaptation of the proposed controller in the sinusoidal experiment.

For the sinusoidal signal experiment studies, the experiment results are depicted in Figs. 11, 12 and 13. The capability estimation of SOB plays a significant role in the efficiency of the suggested method and is provided on Fig. 11. The results show that the variable states can successfully be estimated to facilitate control synthesis. Figures 12a–c displays the output response of the EHSC system, including displacement tracking, the tracking error, and the control action signal, respectively. Overall, Fig. 12 reveals that the proposed method works better than NNPID and ANNBC with the smallest tracking deviation. Meanwhile, the *χ* adaptation of the proposed controller is described in Fig. 13.

**Fig. 14:**
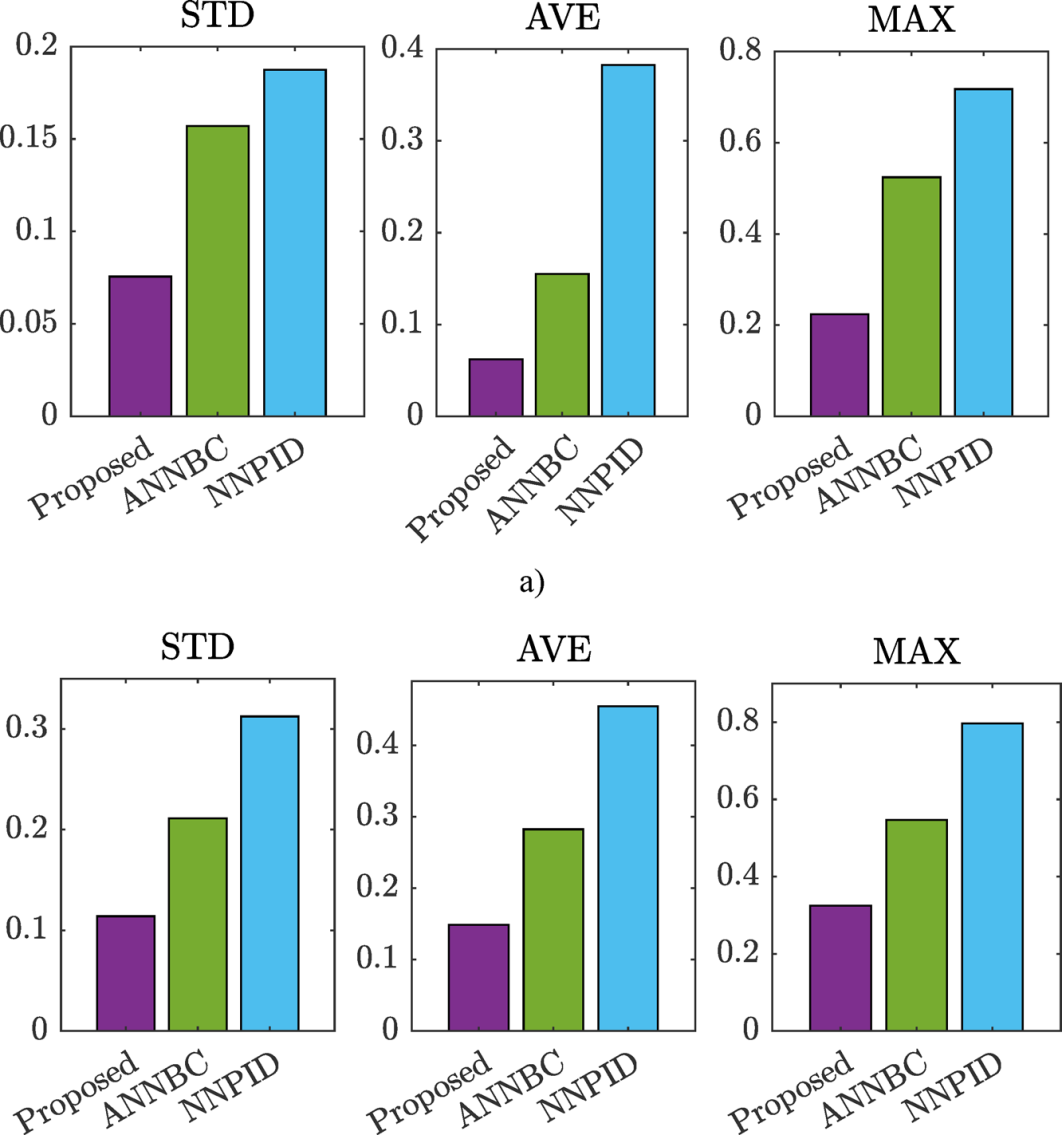
Comparison of performance indexes in the experiment.

To further confirm the merits of the proposed controller, the three performance indicators in the experiment are indicated on Fig. 14. For the ramp experiment, the proposed method achieved a standard deviation (STD) of 0.0756, compared to 0.1571 for ANNBC and 0.1874 for NNPID. The average error (AVE) was 0.0620 for the proposed method, 0.1552 for ANNBC, and 0.3826 for NNPID. The maximum error (MAX) recorded was 0.2237 for the proposed method, 0.5243 for ANNBC, and 0.7175 for NNPID. These results are illustrated in Fig. 14a. In the sinusoidal experiment, the proposed method yielded an STD of 0.1140, whereas ANNBC and NNPID recorded 0.2113 and 0.3124, respectively. The AVE values were 0.1489 for the proposed method, 0.2825 for ANNBC, and 0.4547 for NNPID. The MAX errors were 0.3251 for the proposed method, 0.5465 for ANNBC, and 0.7966 for NNPID. These findings are depicted in Fig. 14b. The above performance indices further demonstrate the efficiency of the proposed methodology, exhibiting the smallest error.

## Conclusion

The control of the EHSC is investigated using an adaptive finite-time backstepping control scheme in the presence of unmeasured states and disturbances. In this paper, a state observer is designed to address the issue of unmeasured states. To handle the unknown system dynamics of the EHSC, the maximum norm neural network (NN) technique is employed to reduce the computational burden caused by the NN training process. Furthermore, an observer-based finite-time backstepping control approach with an adaptation law is developed to enhance operational performance. Finally, both experimental and simulation results on the real EHSC system validate that the proposed control strategy outperforms existing controllers. In the future, event-trigger-based finite-time fuzzy tracking control will be performed to enhance the tracking performance for EHSC with full state constraint.

## Data Availability

The authors confirm that the data supporting the findings of this study are available within the article.
